# 

**Published:** 1889-01

**Authors:** 


					﻿JAJST CTA RY, 1889.
OLD SERIES
Vol. xxv
NEW SERIES
& I855 <>-
Wwa
fflEDIGAMURlWL

fa et	>

^db)
W.F.WESTMORELAND.M D.|
H.V.M.MILLER. M.D.LL .D*
PUBLISHED BY JAMES P.HARRISON&cd
gE______________Atlanta, ga. M
fiiiMMHBViAia ■ »» ■ a a wc An
				

